# Targeting CD83 in mantle cell lymphoma with anti‐human CD83 antibody

**DOI:** 10.1002/cti2.1156

**Published:** 2020-07-15

**Authors:** Ziduo Li, Edward Abadir, Kenneth Lee, Candice Clarke, Christian E Bryant, Wendy Cooper, Geoffrey Pietersz, James Favaloro, Pablo A Silveira, Derek NJ Hart, Xinsheng Ju, Georgina J Clark

**Affiliations:** ^1^ Dendritic Cell Research ANZAC Research Institute Sydney NSW Australia; ^2^ Sydney Medical School The University of Sydney Sydney NSW Australia; ^3^ Institute of Haematology Royal Prince Alfred Hospital Sydney NSW Australia; ^4^ Anatomical Pathology Concord Repatriation General Hospital Sydney NSW Australia; ^5^ Inflammation, Cancer and Infection Burnet Institute Melbourne VIC Australia; ^6^ Baker Heart and Diabetes Institute Melbourne VIC Australia

**Keywords:** antibody–drug conjugate, CD83, immunotherapy, mantle cell lymphoma, NF‐κB

## Abstract

**Objectives:**

Effective antibody–drug conjugates (ADCs) provide potent targeted cancer therapies. CD83 is expressed on activated immune cells including B cells and is a therapeutic target for Hodgkin lymphoma. Our objective was to determine CD83 expression on non‐Hodgkin lymphoma (NHL) and its therapeutic potential to treat mantle cell lymphoma (MCL) which is currently an incurable NHL.

**Methods:**

We analysed CD83 expression on MCL cell lines and the lymph node/bone marrow biopsies of MCL patients. We tested the killing effect of CD83 ADC *in vitro* and in an *in vivo* xenograft MCL mouse model.

**Results:**

CD83 is expressed on MCL, and its upregulation is correlated with the nuclear factor κB (NF‐κB) activation. CD83 ADC kills MCL *in vitro* and *in vivo*. Doxorubicin and cyclophosphamide (CP), which are included in the current treatment regimen for MCL, enhance the NF‐κB activity and increase CD83 expression on MCL cell lines. The combination of CD83 ADC with doxorubicin and CP has synergistic killing effect of MCL.

**Conclusion:**

This study provides evidence that a novel immunotherapeutic agent CD83 ADC, in combination with chemotherapy, has the potential to enhance the efficacy of current treatments for MCL.

## Graphical abstract

Mantle cell lymphoma (MCL) is a blood cell cancer with high mortality. We identified CD83 as a potential therapeutic target. Anti‐CD83 antibody toxin conjugate kills MCL. The combination of chemotherapy with anti‐CD83 antibody could enhance the therapeutic efficacy.

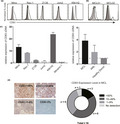

## Introduction

Mantle cell lymphoma (MCL) has an incidence of 0.5 per 100 000 in Western countries and accounts for 2.5–6% of all non‐Hodgkin lymphomas (NHLs).^1^, ^2^ Most MCL cases have an aggressive clinical course and develop resistance to treatment resulting in relapse after a variable period of remission. The median overall survival is 4–5 years.^3^

The MCL cells originate from mature B cells within the mantle zone of lymphatic follicles. The pathogenic hallmark, and potential initiating oncogenic mutation, is the upregulation of cyclin D1 (CCND1) which is predominantly because of the translocation of t (11;14) (q13; q32). In addition, other signalling molecules such as ataxia telangiectasia mutated, members of the nuclear factor κB (NF‐κB) pathway and NOTCH family display constitutive activation in MCL.^4^, ^5^, ^6^

Antibody–drug conjugates (ADCs), which combine monoclonal antibodies (mAbs) with small molecules or toxins, have the advantage of highly specific killing and reduced toxicity than conventional chemotherapy.^7^ The recent approvals of ADC for the treatment of haematological malignancies, such as brentuximab vedotin and inotuzumab ozogamicin,^8^, ^9^ are support for their high efficacy and low toxicity in clinical practice.^10^, ^11^ New ADC in MCL including antibodies targeting novel antigens such as receptor tyrosine kinase‐like orphan receptor 1 (ROR1) and novel payloads for already known targets, such as CD19‐targeted therapy, are under assessment in clinical trials (ClinicalTrials.gov: NCT02669017, NCT02669017 and NCT03424603).

CD83 is a transmembrane immunoglobulin superfamily member with a regulatory function on lymphocyte maturation, activation and homeostasis in the immune system.^12^, ^13^, ^14^ CD83 is expressed by mature dendritic cells and activated lymphocytes but not by other peripheral blood cells.^11^, ^15^ Some tumor cells have high expression of CD83 on the cell surface, such as Hodgkin lymphomas (HLs), diffuse large B‐cell lymphoma, adult acute lymphoblastic leukaemias, gastric extra‐nodal marginal zone lymphomas and lung cancer cells.^15^, ^16^, ^17^, ^18^, ^19^, ^20^ We identified CD83 as a potential therapeutic target in HL, in which an anti‐CD83 ADC effectively killed CD83^+^ HL cells.^21^ Although CD83 is expressed on mature dendritic cells and activated lymphocytes, our data showed that T‐cell immunity is maintained and it does not affect specific T‐cell responses to clinically important viruses after application of anti‐human CD83 antibody *in vitro* and *in vivo*.^22^ The half‐life of the 3C12C antibody in non‐human primates was short, and the administration of anti‐human CD83 antibody is safe in non‐human primates.^21^ A soluble version of CD83 protein (sCD83) with immune suppressive function has been reported. Serum sCD83 concentration is increased in some haematological malignancies, including chronic lymphocytic leukaemia, but returns to normal in patients who responded to chemotherapy.^23^, ^24^ Whether CD83 can be a therapeutic target for MCL has not been investigated. In this study, we analysed the CD83 expression on MCL and tested the ability of CD83 ADC to kill MCL *in vitro* and *in vivo*.

## Results

### CD83 is expressed on some MCL cell lines and in lymph node and bone marrow biopsies from MCL patients

**Figure 1:**
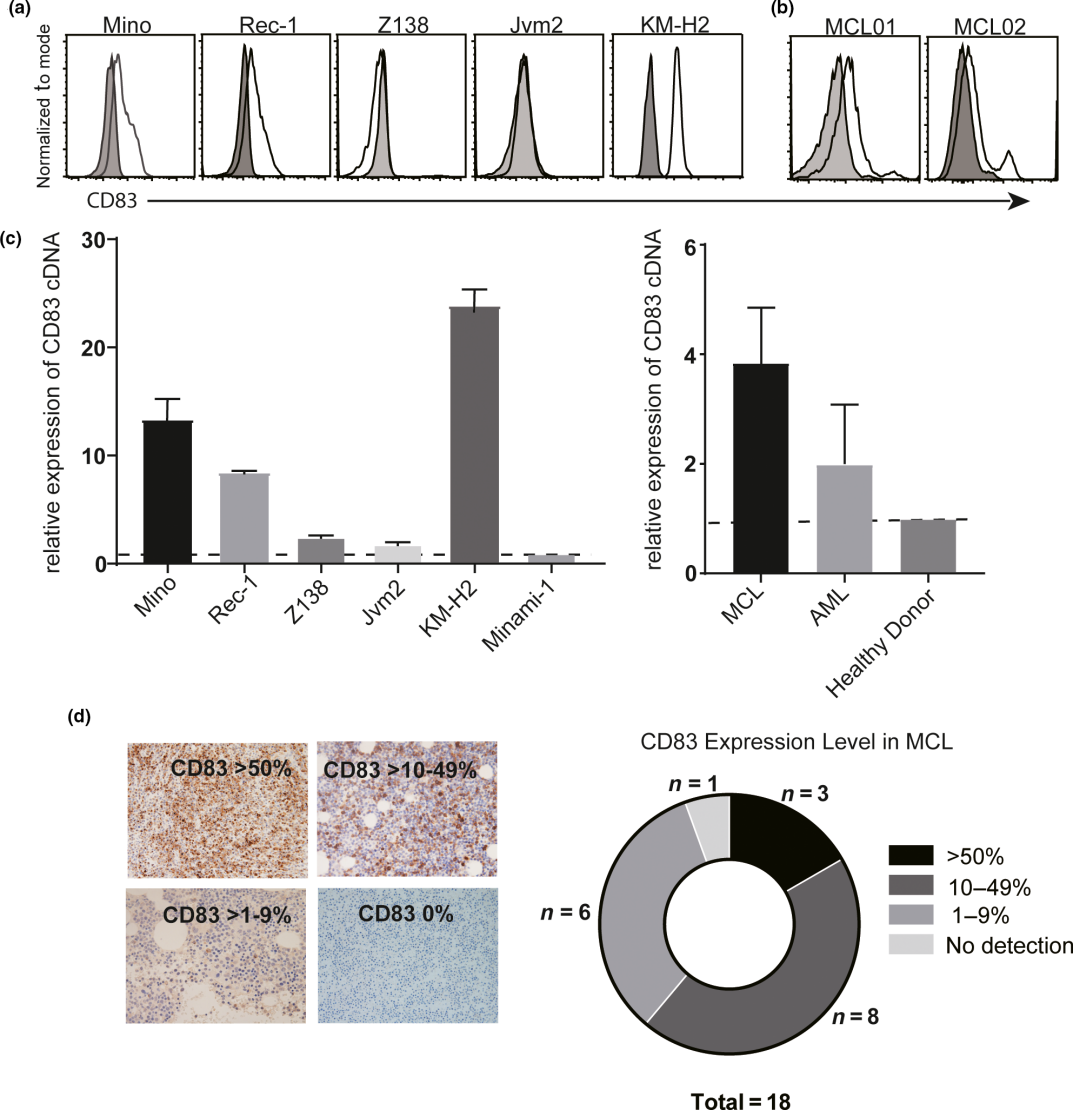
Mantle cell lymphoma (MCL) cell expression of CD83. **(a)** CD83 expression on four MCL cell lines (Mino, Rec‐1, Z138 and Jvm2) was analysed by flow cytometry with human anti‐CD83 antibody 3C12C‐FITC (*n* = 4). **(b)** Primary blastoid non‐nodal MCL patient (MCL01 and MCL02) blood samples were stained with CD19‐V450, CD5‐APC and 3C12C‐FITC and analysed by flow cytometry for the CD83 expression on CD19^+^/CD5^+^ cells. **(c)** Quantitative real‐time PCR analysis of CD83 transcripts in MCL cell lines and MCL primary samples. Data were normalised by the amount of HPRT cDNA and calculated as fold change than CD83 level in Minami‐1 and PBMC from healthy donors, respectively. **(d)** FFPE biopsies from 18 primary MCL patients were stained with anti‐CD83 antibody. Percentages of CD83‐positive cells are shown.

CD83 expression was analysed on MCL cell lines. Mino cells and Rec‐1 cells expressed CD83 on their cell surface, whilst the Z138 and Jvm2 lines did not. The HL cell line KM‐H2 expressed the highest amount of cell surface CD83 (Figure 1a). Cell surface CD83 was detected on the CD19^+^/CD5^+^ population of peripheral blood mononuclear cell (PBMC) from two primary MCL patients, 30.3% CD83^+^ for MCL01 and 19.1% for MCL02 (Figure 1b). As expected, CD83 mRNA transcripts were more abundant in CD83^+^ MCL lines (Mino and Rec‐1) than CD83^−^ MCL cells (Z138 and Jvm2). Similarly, primary MCL cells expressed more CD83 mRNA transcripts than primary acute myeloid leukaemia cells or healthy donor PBMC (Figure 1c).

CD83 expression was analysed on either formalin‐fixed paraffin‐embedded (FFPE) lymph node or bone marrow biopsies from 18 MCL patients. Patients' clinical characteristics are detailed in Table 1. The average age of patients was 65.7 years, and most samples were from men (16/18, 88.9%). 83.3% (15/18) of the patients were assessed to be stage IV based on Ann Arbor Stage criteria.^25^ The Average Mantle Cell Lymphoma International Prognostic Index (MIPI) scores of high risk, intermediate or low risk were 55.6%, 22.2% and 22.2%, respectively. Among the 18 patients, 3/18 (16.67%) biopsies expressed high levels of CD83 (> 50% positive) on the MCL cells, 8/18 (44.44%) expressed moderate levels (10–50% positive), 6/18 (33.33%) expressed low levels of CD83 (< 10% positive) and 1/18 (5.55%) had no CD83 detection (Figure 1d). The intensity of CD83 staining on biopsies was analysed; CD83 staining was very strong (+++) in 9/18 (50%), medium level (++) in 2/18 (11.1%), weak level (+) in 6/18 (33.3%) and no CD83 staining in 1/18 (5.55%). There is no correlation of CD83 expression level (cut‐off 10% as low/high) with MIPI score or clinical stage.

**Table 1:** Characteristics of 18 patients with mantle cell lymphoma

Age at enrolment (mean ± s.d.)	65.7 ± 8.7 years
Sex	
Male; *n* (%)	16 (88.9)
Female; *n* (%)	2 (11.1)
Histologic subtype *n* (%)	
Typical	16 (88.9)
Blastoid	2 (11.1)
Ann Arbor Stage *n* (%)	
I/III	2 (11.1)
VI	15 (83.3)
Unidentified	1 (5.6)
Mantle Cell Lymphoma International Prognostic Index *n* (%)	
High risk	10 (55.6)
Intermediate risk	4 (22.2)
Unidentified	4 (22.2)
Medium survival after diagnosis (years)	6 years

### Anti‐CD83 antibody–drug conjugate kills CD83^+^ MCL cells *in vitro*

**Figure 2:**
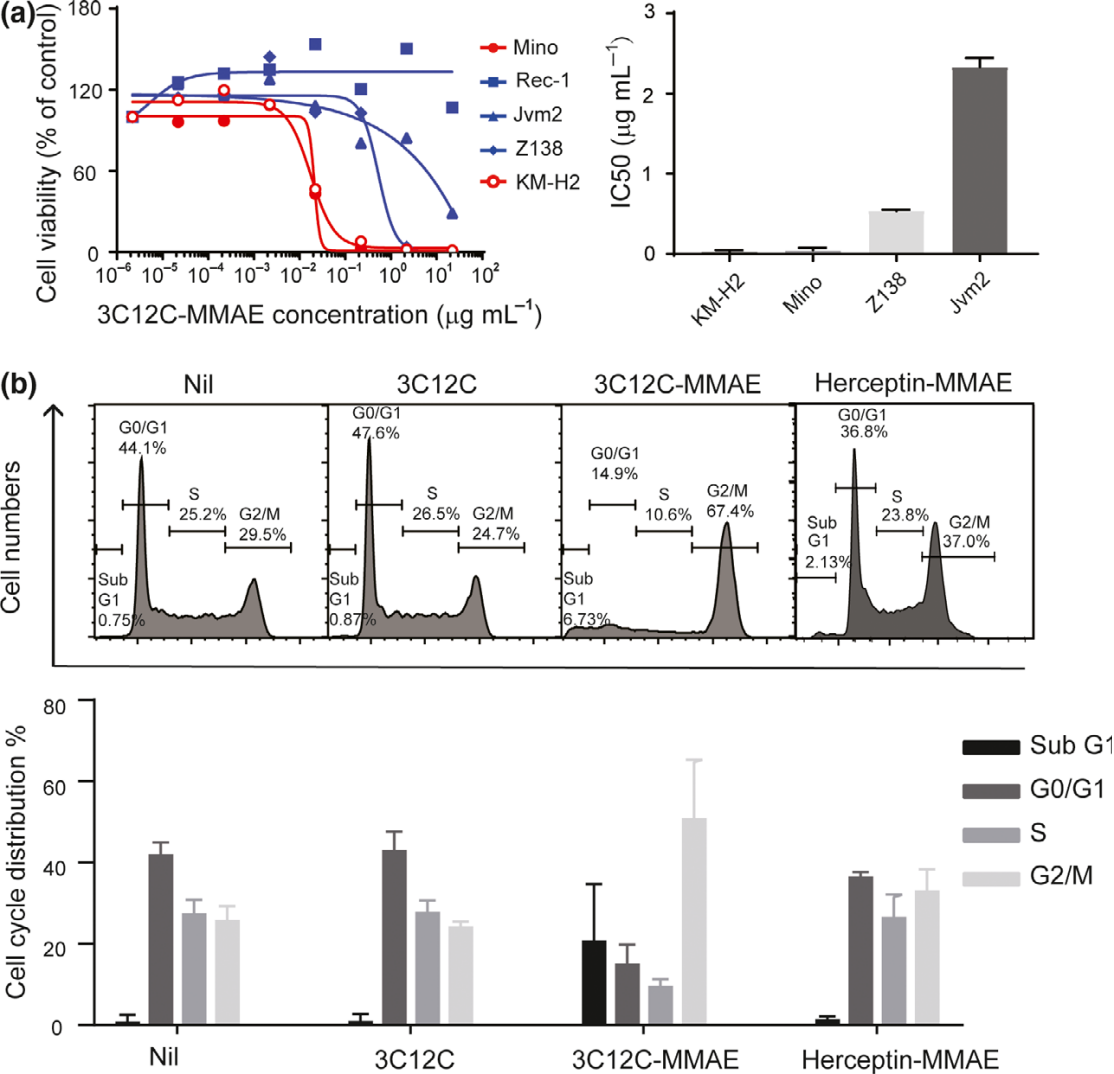
3C12C conjugation with monomethyl auristatin E (3C12C‐MMAE) kill mantle cell lymphoma (MCL) lines *in vitro*. **(a)** CD83^+^ KM‐H2, Mino, Rec‐1 or CD83^−^ Z138, and Jvm2 cells were cultured with different concentrations of 3C12C‐MMAE for 72 h before determining viable cells by CellTiter‐Glo Luminescent Cell Viability assay. Data are from one representative experiment (left), and the mean ± SEM of half‐maximal inhibitory concentration (IC50) from three experiments (right) is shown. **(b)** Mino cells were cultured in 3C12C‐MMAE (antibody concentration of 0.2 µg mL^−1^) for 18 h, and then, the cells were fixed for cell cycle analysis by flow cytometry. Data are from one representative experiment (upper panel), and the mean ± SEM of three experiments (bottom panel) is shown.

The anti‐CD83 ADC (3C12C‐MMAE) drug activity was tested on four MCL lines (Mino, Rec‐1, Z138 and Jvm2). CD83^+^ HL cell line KM‐H2 was included as control. Cells were exposed to 3C12C‐MMAE for 72 h, and then, the metabolic‐based luciferase assay CellTiter‐Glo was used to quantify viable cells. 3C12C‐MMAE killed MCL CD83^+^ cells and Mino cells efficiently. Although Mino cells express less cell surface CD83 than KM‐H2 cells, the IC50 value of both cell lines is similar: 0.017 and 0.021 µg mL^−1^ for KM‐H2 and Mino, respectively (Figure 1a). Both the CD83^+^ Mino and Rec‐1 cells were susceptible to naked 3C12C via NK‐mediated ADCC in dose‐dependent manner (Supplementary figure 1). Rec‐1 had a higher IC50 (> 10 µg mL^−1^) when exposed to 3C12C‐MMAE than Mino cells. The resistance of Rec‐1 to MMAE was confirmed by culturing MCL lines with MMAE (Supplementary figure 2). Since MMAE‐conjugated antibody has been reported to induce G2/M phase arrest,^26^ we tested the effect of 3C12C‐MMAE on the cell cycle of CD83^+^ Mino cells. About 45% of Mino cells were arrested in G2/M phase after 18‐h exposure to 3C12C‐MMAE, and this was accompanied by a concomitant decrease in cells in G0/G1 phase. The control ADC, Herceptin‐MMAE, did not change the cell cycle of Mino cells (Figure 2b).

### Anti‐CD83 antibody–drug conjugate effectively kills MCL in a xenograft model

**Figure 3:**
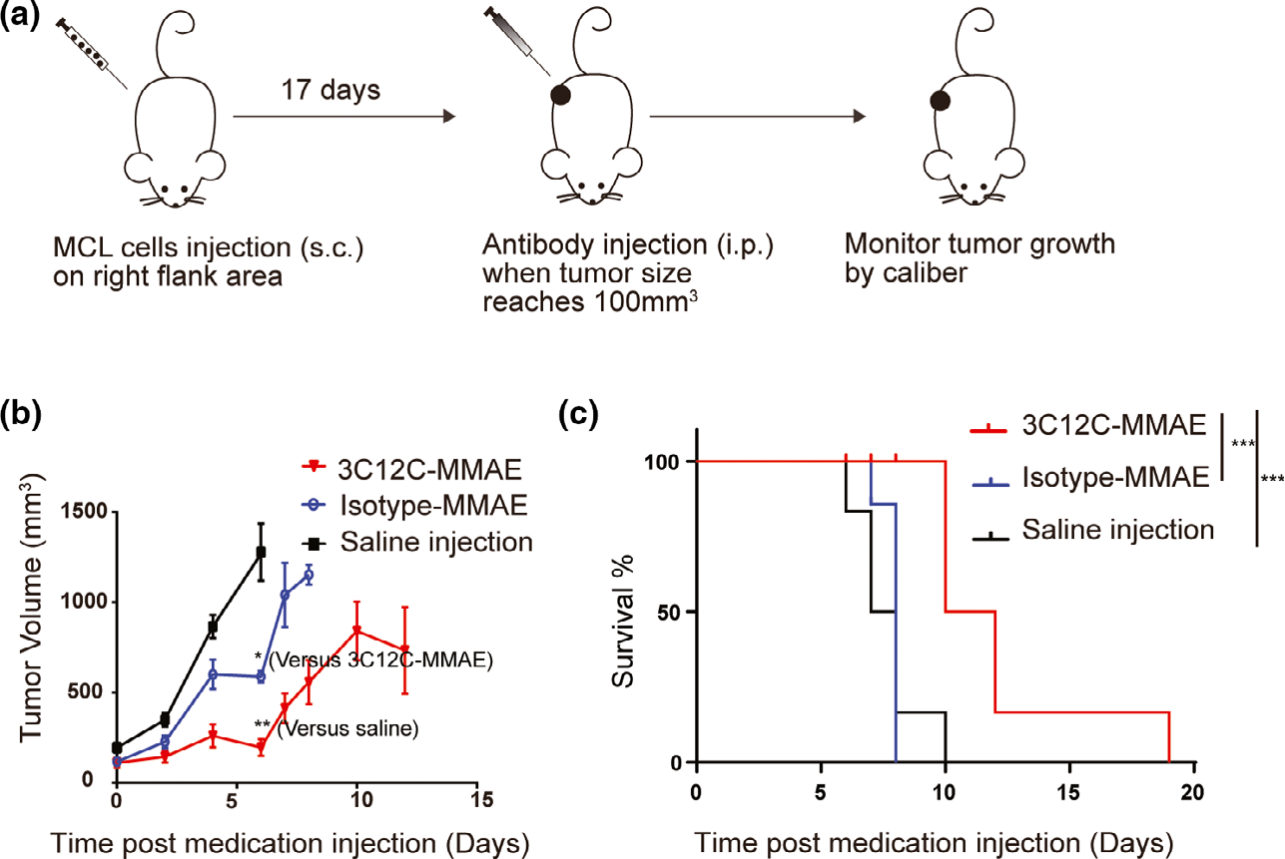
CD83 ADC kills MCL in xenograft mice. **(a)** Schematic design of the MCL xenograft NSG mouse model. **(b)** Quantification of tumor volume from MCL engrafted mice treated with 3C12C‐MMAE, isotype control‐MMAE or saline was shown (*n* = 6 mice per group). One of three representative experiments is shown. **(c)** Kaplan–Meier survival curves of mice implanted with Mino cells (*n* = 6 mice per group). Data were analysed with a log‐rank test. ***P < 0.001. One of three representative experiments is shown.

To evaluate the efficacy of our ADC *in vivo*, we established a xenograft mouse model in which NSG mice were subcutaneously injected with Mino cells (Figure 3a). On day 18 post‐injection of Mino cells, when tumors were palpable, 3C12C‐MMAE or control vehicle was injected intraperitoneally. Mice were euthanised at day 6 post‐injection of control vehicle, and engrafted tumor cells were harvested for flow cytometry analysis. Engrafted tumors were CD5^+^CD19^+^ and expressed a similar amount of CD83 as the *in vitro*‐cultured Mino cells (Supplementary figure 3). 3C12C‐MMAE inhibited the tumor growth and increased the survival of tumor engrafted mice than control antibody–MMAE conjugate (Figure 3b and c).

### CD83 upregulation correlates with NF‐κB activation in MCL

**Figure 4:**
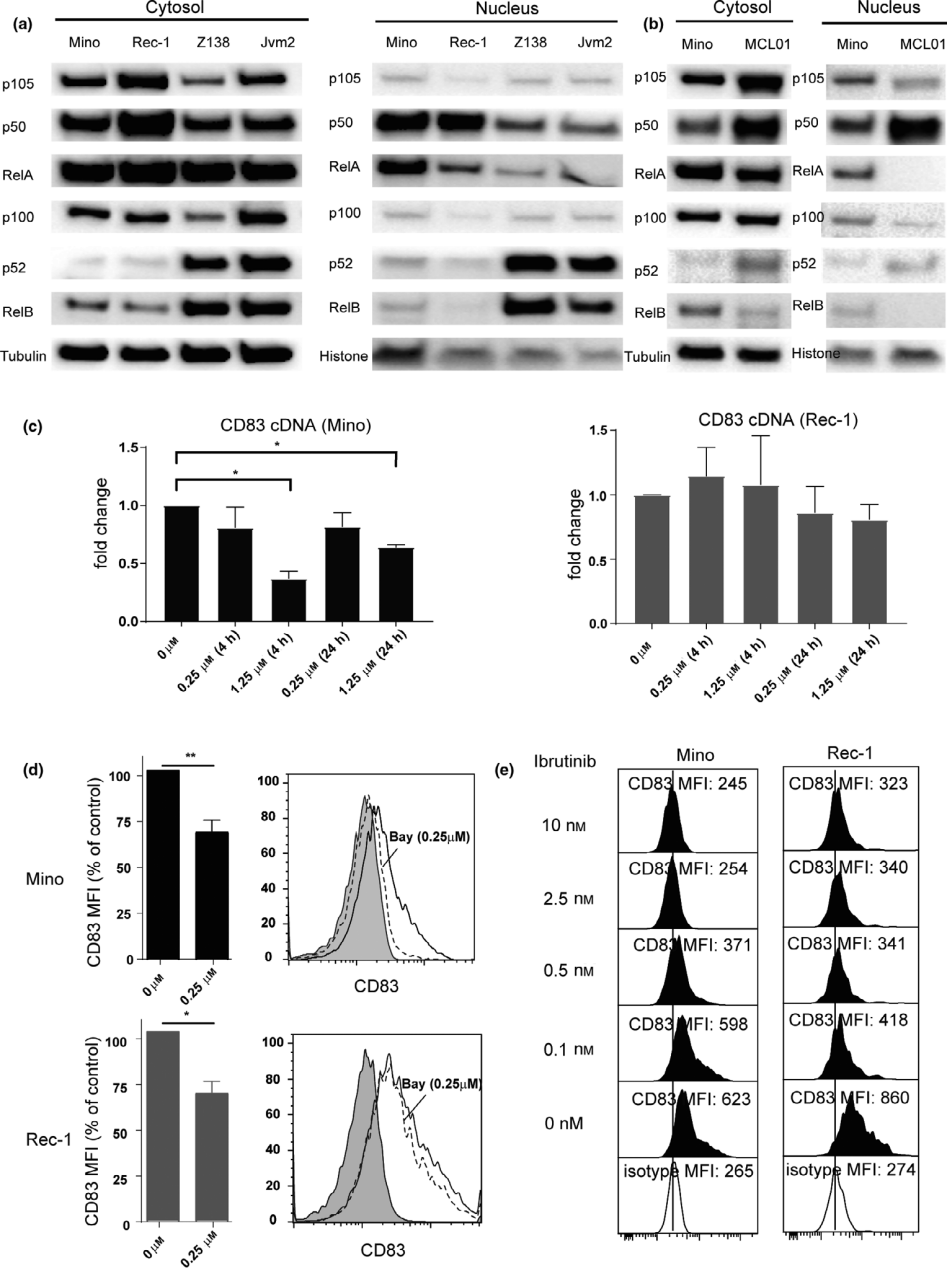
Activation of NF‐κB increases CD83 expression in MCL. **(a)** Western blot analysis of canonical pathway NF‐κB protein (p105/p50 and RelA) and non‐canonical pathway NF‐κB protein (p100/p52 and RelB) levels in the cytosolic and nuclear extracts of CD83^+^ MCL cells (Mino and Rec‐1) and CD83‐ MCL cells (Z138 and Jvm2). **(b)** Western blot analysis of canonical NF‐κB protein (p105/p50 and RelA) and non‐canonical NF‐κB protein (p100/p52 and RelB) levels in the cytosolic and nuclear extracts of CD83^+^ MCL cells (Mino) and primary MCL cells (MCL01). **(c)** CD83^+^ cells were treated with either a DMSO control or canonical NF‐κB inhibitor BAY‐11‐7082 at different concentrations (0.25 or 1.25 µm) for 4 or 24 h. Real‐time PCR (qPCR) analyses of CD83 cDNA from Mino (left) and Rec‐1 (right) cells are shown. *P < 0.05. **(d)** CD83^+^ cells were treated with either a DMSO control or BAY‐11‐7082 (0.25 µm) for 24 h. Cell surface CD83 expression on Mino (upper) and Rec‐1 (bottom) was analysed by flow cytometry, and mean fluorescence intensity (MFI) normalised to the untreated samples is shown (*n* = 3). *P < 0.05; **P < 0.01. Data from one representative experiment are shown in right panel. Filled histogram: isotype control. Open histogram with solid line: CD83 staining on cells treated with DMSO. Open histogram with dash lines: cells treated with BAY‐11‐7082 (0.25 µm). **(e)** Mino cells or Rec‐1 cells were cultured in the different concentration of ibrutinib for 72 h, CD83 expression was analysed by flow cytometry with human anti‐CD83 antibody 3C12C‐FITC, and mean fluorescent intensity of one representative experiment (*n* = 3) is shown.

The *CD83* promoter contains NF‐κB‐binding sites. Activation of NK‐κB in normal B cells and some B‐cell malignancies induces CD83 expression.^27^, ^28^ It has been reported that the canonical NF‐κB pathway is activated in some MCL cell lines and primary samples.^5^, ^29^ To reveal the potential relationship between CD83 expression and NF‐κB activation status in MCL, we extracted cytosol and nuclear protein from MCL cell lines and analysed NF‐κB activation by Western blot. Although the activation of NF‐κB in both CD83^+^ and CD83^−^MCL lines was detected, CD83^+^ MCL cells, Mino and Rec‐1, showed elevated p50 and RelA in the nuclear fraction, indicating strong canonical NF‐κB pathway activation. In CD83^−^ cell lines, p52 and RelB levels were high in the cytosol and nuclear fractions indicating non‐canonical NF‐κB pathway activation (Figure 4a). The primary MCL PBMC cell lysate (MCL01) had a similar canonical NF‐κB pathway activation pattern to Mino cells (Figure 4b). We then exposed CD83^+^ cells to the canonical NF‐κB pathway inhibitor, BAY11‐7082. CD83 mRNA transcripts were reduced in both Mino and Rec‐1 cells exposed to 1.25 µm BAY11‐7082 for 18 h (Figure 4c). CD83 cell surface protein was also reduced by canonical NF‐κB inhibitors (Figure 4d). Ibrutinib, a reagent for the treatment of refractory and relapsed MCL, blocks activity of a specific protein called Bruton's tyrosine kinase (BTK) and NF‐κB signalling. Our data showed it downregulated CD83 expression on MCL cell lines (Mino and Rec‐1) and neutralised the killing effect of 3C12C‐MMAE on Mino (Figure 4e and Supplementary figure 4).

### CD83 ADC has a synergic killing effect with anthracyclines and alkylating agents

**Figure 5:**
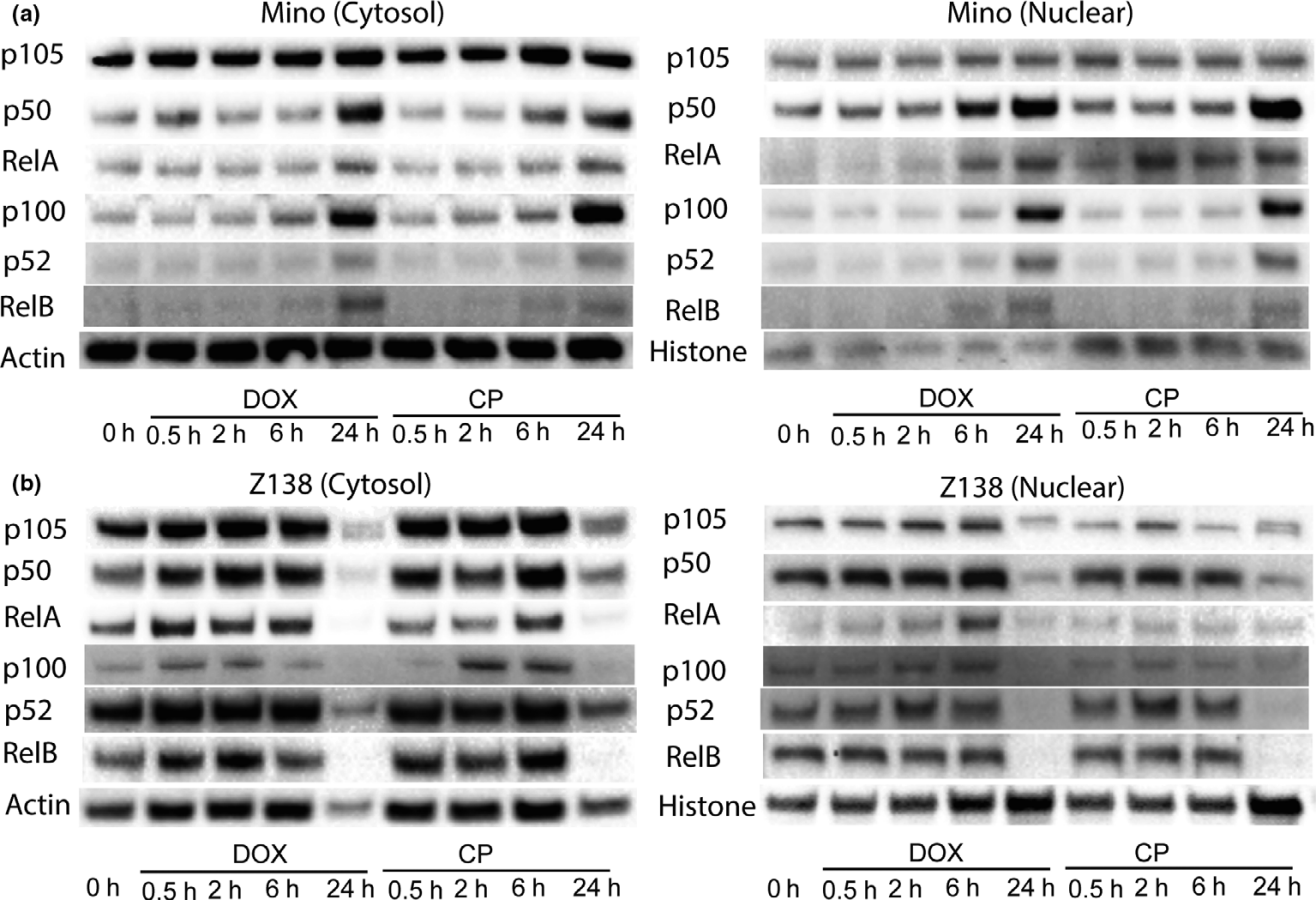
Doxorubicin and cyclophosphamide activated NF‐κB in MCL lines. Mino or Z138 cells were cultured in the presence of DOX (0.2 μg mL^−1^) or CP (0.5 mg mL^−1^) for 30 min, 2 h, 6 h and 24 h. Cytoplasmic and nuclear proteins were isolated. Immunoblot analysis of cell lysate was performed with anti‐NF‐κB antibodies.

**Figure 6:**
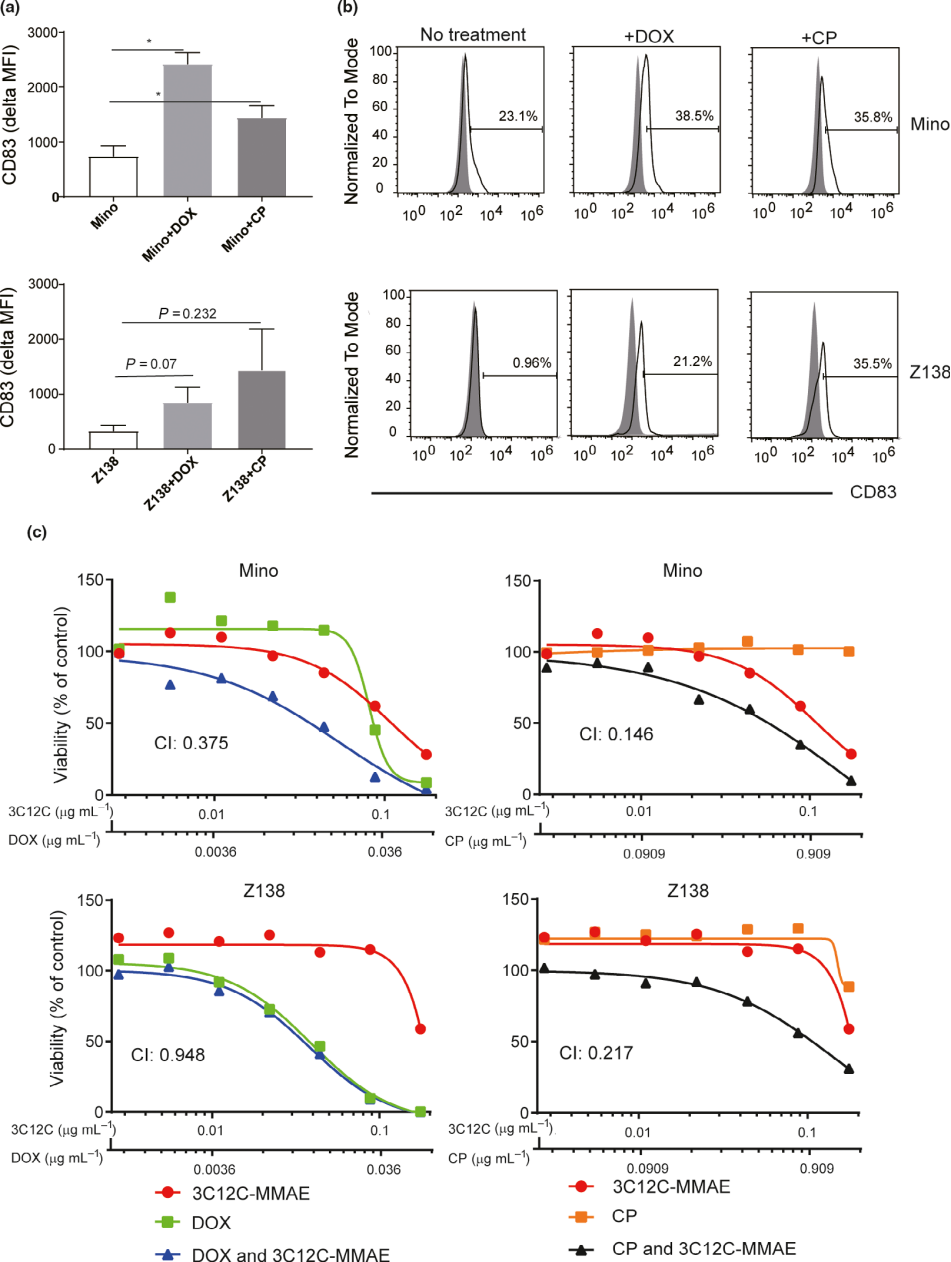
3C12C ADC synergised with doxorubicin/cyclophosphamide. Mino or Z138 cells were cultured in the presence of DOX (0.02 µg mL^−1^) or CP (0.2 mg mL^−1^) for 48 h, and cell surface CD83 expression was analysed by flow cytometry. Mean fluorescent intensity ± SEM of three experiments **(a)** and data from one of three experiments **(b)** are shown. **(c)** Mino or Z138 cells were cultured with serially diluted 3C12C‐MMAE (0.176, 0.088, 0.044, 0.022, 0.011, 0.0055, 0.00275 µg mL^−1^), DOX (0.064, 0.032, 0.016, 0.008, 0.004, 0.002, 0.001 µg mL^−1^), CP (1.6, 0.8, 0.4, 0.2, 0.1, 0.05, 0.025 µg mL^−1^) or the combination of 3C12C‐MMAE/DOX (0.176/0.064, 0.088/0.032, 0.044/0.016, 0.022/0.008, 0.011/0.004, 0.0055/0.002, 0.00275/0.001 µg mL^−1^), and the combination of 3C12C‐MMAE/CP (0.176/1.6, 0.088/0.8, 0.044/0.4, 0.022/0.2 0.011/0.1, 0.0055/0.05, 0.00275/0.025 µg mL^−1^) for 72 h. CellTiter‐Glo Luminescent cell viability assay was used to determine the killing effect. Data are from one of three independent experiments. The Combination Index (CI) was analysed with CompuSyn.

Nuclear factor‐κB activation can be induced in some malignancies by cytotoxic medications such as doxorubicin (DOX).^30^ Mino and Z138 cells were cultured with drugs included in standard chemotherapy regimens and novel treatment agents (Supplementary table 1). Both canonical (p105/p50, RelA) and non‐canonical NF‐κB (p100/p52 and RelB) were increased in CD83^+^ Mino cells, especially in the nuclear fraction, after treatment with DOX or cyclophosphamide (CP). CD83^−^ Z138 cells were more sensitive to death from exposure to DOX and CP and upregulated NF‐κB molecules after short culture with DOX or CP (Figure 5 and Supplementary figure 5). Furthermore, DOX and CP upregulated CD83 on CD83^+^ Mino and CD83^−^ Z138 cells (Figure 6a and b), whilst other chemotherapy drugs had no effect on CD83 expression (Supplementary table 1). This leads us to test the combination effect of 3C12C‐MMAE with some chemotherapies. 6c). Whilst the CI of Z138 with 3C12C‐MMAE and DOX was close to one, in three experiments it was consistently less than one.

## Discussion

CD83 expression has been reported in some lymphomas and leukaemias.^21^, ^23^, ^24^ We showed here that CD83 is upregulated in 50% of primary MCL samples and some MCL cell lines. Interestingly, the immunohistochemical staining of primary MCL cells showed a pattern of heterogeneous expression, which reflects the heterogeneity of sub‐clonal populations within the MCL tumor tissue, which has been reported by others.^31^ We observed CD83 upregulation by DOX and CP in MCL which aligns with NF‐κB activation induced by the DNA damage‐independent stress from chemotherapy.^32^, ^33^, ^34^

We tested anti‐CD83 Ab as a single ADC agent in the treatment of CD83^+^ MCL *in vitro* and in a xenogeneic mouse model. Interestingly, although CD83 expression in Mino cells is not as high as that on the classical Hodgkin lymphoma cells, KM‐H2, they have a similar sensitivity to the anti‐CD83 ADC. This could be the hyper‐sensitivity of Mino cells to toxin MMAE and/or fast internalisation of anti‐CD83 Ab by MCL cells. Similar phenomena have been observed with ADC targeting cells with low antigen expression.^7^ Kovtun *et al*.^35^ found that ADC killed not only the target antigen‐positive cells but also the neighbouring antigen‐negative cells which depended on the nature of the reducible disulphide bond linker and the release of the payload into adjacent antigen‐negative cells. An important factor that affects naked therapeutic antibody efficacy is the expression level of targeted antigen. ADCs have shown to be more effective than naked antibodies over a wider range of antigen expression levels.^7^ For example, whilst CD33‐positive acute myelogenous leukaemia tumors express relatively low levels of target antigen (5000–10 000 receptors per cell), an ADC that targets the CD33 antigen still shows meaningful clinical response rates.^36^ Similarly, an anti‐CD83 ADC has the potential to be effective in a substantial proportion of MCL. Even if 40% of biopsies express minimal or no CD83, this killing effect of anti‐CD83 ADC will be increased by concurrent administration of chemotherapy drugs that increase CD83 expression in MCL (see below). Although CD83^+^ Rec‐1 cells could be killed by CD83 Ab via ADCC, similar to KM‐H2 and Mino cells in a dose‐dependent manner (Supplementary figure 1), they are not sensitive to 3C12C‐MMAE which is likely because of their resistance of MMAE.^37^

Current treatment of younger patients with MCL often includes DOX and CP. Given that these drugs were capable of inducing CD83 expression in MCL, and our data showed the synergistic effect of anti‐CD83 ADC with a conventional chemotherapy (DOX and CP), these patients may benefit from combination regimens of chemotherapy and anti‐CD83 ADC. Whilst treating younger patients with a therapy that targets mature dendritic cells may raise concerns that host immunity will be compromised, in our earlier studies in mice and non‐human primates we found that there was little 3C12C antibody 10 days after immunisation. The short half‐life of the antibody would limit long‐term effects on prospective immune challenges. Ibrutinib is used to treat relapsed and refractory MCL by inhibiting Bruton's tyrosine kinase and NF‐κB signalling.^6^, ^38^ Ibrutinib also inhibits NF‐κB pathway. It significantly decreased tumor proliferation and expression of surface activation markers CD69 and CD86 in chronic lymphocytic leukaemia which has similar clinical manifestations to MCL.^39^ Our data showed that ibrutinib reduced the CD83 expression and neutralised the killing of anti‐CD83 ADC on MCL. This further supports the effect of anti‐CD83 ADC in MCL via activating of NF‐κB but highlights that ibrutinib should not be combined with anti‐CD83 ADC clinically.

Mantle cell lymphoma is a group of mature B‐cell lymphomas comprising two possible molecular pathogeneses. The pre‐malignant B cells within the classic and most common subgroup of MCL originate from mantle zone and do not enter germinal centres. This subgroup does not have hypermutated Ig but has SOX11^+^. The pre‐malignant cells of the minor subgroup enter into the germinal centre. These cells have hypermutated Ig and are SOX11^−^.^40^ From limited sample size, we could not determine the correlation between CD83 expression and SOX11 expression on MCL. The CD83 gene promoter contains NF‐κB‐binding sites.^27^, ^41^ Several types of lymphomas, such as HL, diffuse larger B‐cell lymphoma and chronic lymphocytic lymphoma, which have been reported as CD83^+^, also showed NF‐κB activation as a hallmark in their pathogenesis.^42^, ^43^, ^44^, ^45^ MCL depends on NF‐κB signalling for continued growth and proliferation and showed distinct NF‐κB activation.^5^, ^6^ Our results showed that CD83 expression correlated with canonical NF‐κB activation in MCL.

The prospects for targeting CD83 on MCL patients requires identification of CD83^+^ MCL patients, application of the most recent ADC techniques to generate anti‐CD83 ADC and the combination of ADC with current chemotherapy. In addition, targeting multiple pathways including the NF‐κB pathway potentially overcomes resistance as a result of targeting a single pathway to treat MCL. In conclusion, our research demonstrated CD83 expression in MCL cell lines and primary tumor cells and showed the anti‐CD83 ADC is a possible therapy for CD83^+^ MCL treatment.

## Methods

### Patient sample collection

Mantle cell lymphoma patient samples were collected with informed consent approved by the Sydney Local Health District Human Research Ethics Committee (X15‐0464&LNR15/RPAH/615), consistent with the declaration of Helsinki. Archival FFPE lymph node or bone marrow biopsies were obtained from 18 MCL patients at initial diagnosis (Table 1). Fresh PBMCs were purified by Ficoll‐Hypaque (GE Healthcare, Uppsala, Sweden) density gradient from two MCL patients' blood samples (MCL01 and MCL02) collected at initial diagnosis and relapse, respectively.

### Human anti‐CD83 antibody and antibody–drug conjugation

Human anti‐CD83 antibody 3C12C and 3C12C‐monomethyl auristatin E (MMAE) conjugate were produced in house as described.^21^, ^46^

### Cell culture

Mantle cell lymphoma cell lines (Mino, Rec‐1, Jvm2, Z138, purchased from ATCC, Manassas, VA, USA), HL cell line KM‐H2 (gift from Prof Volker Diehl, University of Cologne, Germany) and the erythroleukaemia cell line HEL (purchased from ATCC) were cultured in complete RPMI‐1640 medium containing 10% human foetal calf serum, 2 mm GlutaMAX™, 100 U mL^−1^ penicillin, 100 µg mL^−1^ streptomycin, 1 mm sodium pyruvate, 10 mm HEPES and 10 µm β mercaptoethanol (Thermo Fisher Scientific, Scoresby, VIC, Australia) at 37°C, in 5% CO_2_. BAY11‐7082 (Sigma‐Aldrich, St. Louis, MO, USA), ibrutinib (Selleckchem, Houston, TX, USA), doxorubicin (DOX; DBL Pharmaceuticals, Dhaka, Bangladesh) and CP (Slade Health, Mount Waverley, Vic, Australia) were added to cell cultures as described.

### Immunohistochemical staining

Immunohistochemical staining was performed on 3‐µm sections of FFPE biopsy tissue of lymph node or bone marrow from MCL patients as described previously.^21^, ^46^ The primary CD83 antibody used was the F5 clone (Santa Cruz Biotechnology, Dallas, TX, USA). Staining was performed on a Leica Bond III Autostainer (Leica Biosystems, Wetzlar, Germany) using a Bond Polymer Refine Detection Kit for visualisation with 3,3′‐diaminobenzidine (DAB).

### Flow cytometry

The following antibodies were used: anti‐human CD5‐APC (clone L17F12; BD Biosciences, Franklin Lakes, NJ, USA.), anti‐human CD19‐PE (clone HIB19; BD Biosciences), anti‐human CD45‐AF488 (clone HI30; BioLegend, San Diego, CA, USA.), anti‐mouse CD45‐PerCP Cy5.5 (clone 30‐F11; BD Biosciences,), anti‐human CD83‐FITC (clone HB15a; Beckman and Coulter, Brea, CA, USA) or 3C12C‐FITC (made in house).^22^ Data were collected using a Fortessa X‐20 or Accuri C6 (BD Biosciences) flow cytometer, and the data were analysed with FlowJo software (Treestar, San Carlos, CA, USA).

### Cell viability assays and cell cycle measurement

Cells (5000 per well) were cultured for 72 h in 200 µL complete RPMI‐1640 medium containing various concentrations of 3C12C‐MMAE, DOX, CP alone or combined. The cell viability was analysed with the CellTiter‐Glo Luminescent Cell Viability Kit according to the manufacturer's instruction (Promega, Madison, WI, USA), and half‐maximal inhibitory concentration (IC50) was calculated. For cell cycle measurement, cells were cultured in complete RPMI‐1640 medium containing 3C12C, 3C12C‐MMAE or irrelevant Ig‐MMAE (Herceptin‐MMAE‐conjugated in house using the same chemistry as 3C12C‐MMAE) for the indicated times and then cells were fixed in 70% cold ethanol for 2 h. Cells were stained with propidium iodide (PI) in the presence of 0.2 mg mL^−1^ DNase‐free RNase A (Sigma‐Aldrich) and analysed by flow cytometry.

### Western blots

Mantle cell lymphoma cell lines and cells from one primary MCL sample treated with or without DOX (0.2 µg mL^−1^) and CP (0.2 mg mL^−1^) for different time points were lysed, and the nuclear and cytoplasmic fractions were isolated using NE‐PER Kit (Thermo Fisher Scientific) according to the manufacturer's instruction. The protein concentration of each fraction was measured using a BCA Protein Assay Reagent Kit (Thermo Fisher Scientific). Five to ten microgram of protein was separated by SDS‐PAGE through a 4–12% Bis‐Tris Plus gel (Thermo Fisher Scientific) and transferred onto nitrocellulose membranes using an iBlot blotting system (Thermo Fisher Scientific). Following blocking with 5% bovine serum albumin (BSA) in Tris‐buffered saline (TBS) for 2 h at room temperature and washing with 0.1% Tween 20 TBS (TTBS), the membranes were incubated overnight at 4°C with primary antibody in TTBS containing 5% BSA. The primary antibodies included rabbit anti‐p105/p50 (ab32360: Abcam, Cambridge, UK), mouse anti‐p100/p52 (05‐361: EMD Millipore, Burlington, MA, USA), rabbit anti‐p65 (8242S: Cell Signalling Technology, Danvers, MA, USA), rabbit anti‐RelB (ab18027; Abcam), rabbit anti‐alpha‐Tubulin (T6074; Sigma‐Aldrich), mouse anti‐human beta‐actin (Bio‐Rad, Hercules, CA, USA) and rabbit anti‐human histone (#9715; Cell Signalling Technology). Secondary anti‐mouse, or anti‐rabbit, antibody–horseradish peroxidase conjugate (1:3000 dilution) was incubated with membranes for 1 h at room temperature and washed with TTBS. The blots were detected with the enhanced chemiluminescent (Bio‐Rad,) reagents on a ChemiDoc MP imaging system (Bio‐Rad) according to the manufacturer's instructions. The relative protein expression level was analysed with Image Lab 4.1 Software (Bio‐Rad).

### MCL xenograft mouse tumor model

Five‐ to six‐week‐old female NOD.Cg‐*Prkdc^scid^Il2rg^tm1Wjl^*/SzJ (NSG) mice were purchased from Australian Bioresources and housed under specific pathogen‐free conditions. Experimental procedures were approved by the Sydney Local Health District) animal welfare committee. Each mouse received 5 × 10^6^ Mino cells injected subcutaneously on right flank area. On day 18 post‐cell injection, mice were treated with a single dose of 3C12C‐MMAE (2.5 mg kg^−1^), Herceptin‐MMAE (2.5 mg kg^−1^) or saline only intraperitoneally. Mice were monitored for tumor growth by measuring the tumor volume with digital callipers (tumor volume = 1/2(length × width^2^)). Following euthanasia, the engrafted tumors were harvested, passed through a 100‐µm nylon cell strainer (BD Biosciences) to prepare a single‐cell suspension, washed with PBS and analysed by flow cytometry.

### Statistical analysis

Statistical analyses were performed using Prism 6.0 (GraphPad Software, San Diego, CA, USA). Standard error of the mean is shown unless otherwise stated. The unpaired two‐tailed Student's *t*‐test or log‐rank (Mantel–Cox) test were used as described. *P*‐values < 0.05 were considered significant. *P < 0.05; **P < 0.01; and ***P < 0.001. The CI was analysed with CompuSyn (ComboSyn, Inc., Paramus, NJ, USA).^47^

## Conflict of Interest

GJC is a director of DendroCyte BioTech Pty Ltd and Kira Biotech Pty Ltd. There are no other conflicts of interest to disclose.

## Author contributions

ZL and XJ: Experimental design and performance of the experiments, data analysis and writing of the manuscript. DNJH and GJC: Experimental design, data analysis and editing of the manuscript. EA, CB and WC: Patient recruitment. KL and CC: Performance and interpretation of the immunohistochemistry staining. JF: Flow cytometry on clinical samples. GP: Design and preparation of 3C12C‐MMAE and Herceptin‐MMAE. PAS: Mouse experiments and editing of the manuscript.

## Supporting information

Supplementary Material
