# Supplementary material for: Targeting CD83 in mantle cell lymphoma with anti‐human CD83 antibody
Source: Clin Transl Immunology. 2020 Jul 15;9(7):e1156. doi: 10.1002/cti2.1156 (PMC7362189; doi:10.1002/cti2.1156)
Supplement: Supplementary file 1 — Supplementary Material [file CTI2-9-e1156-s001.pdf]

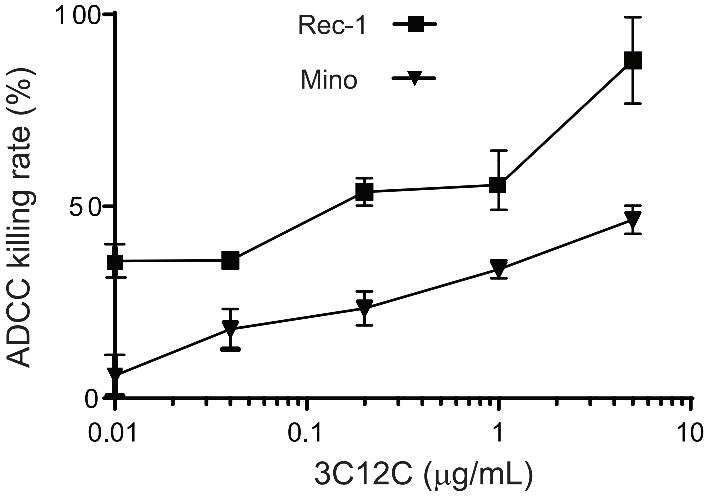

Supplementary figure 1

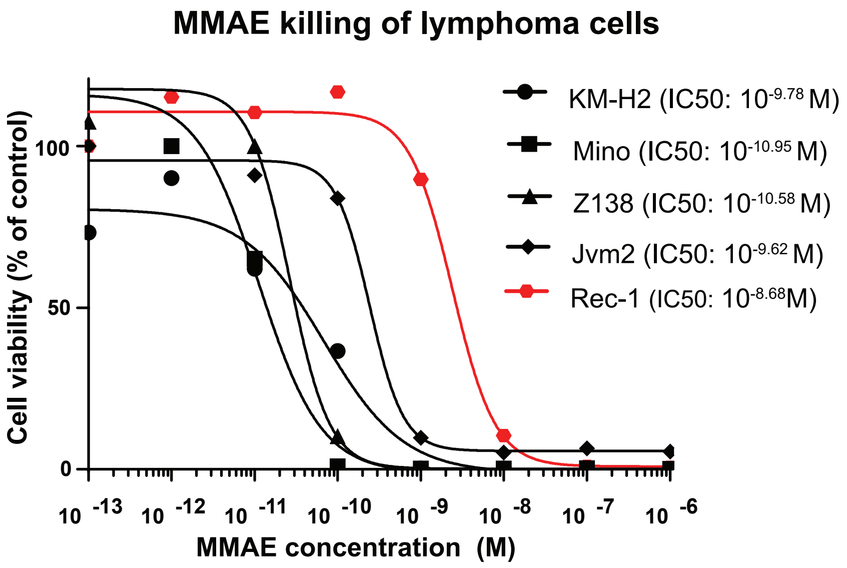

Supplementary figure 2

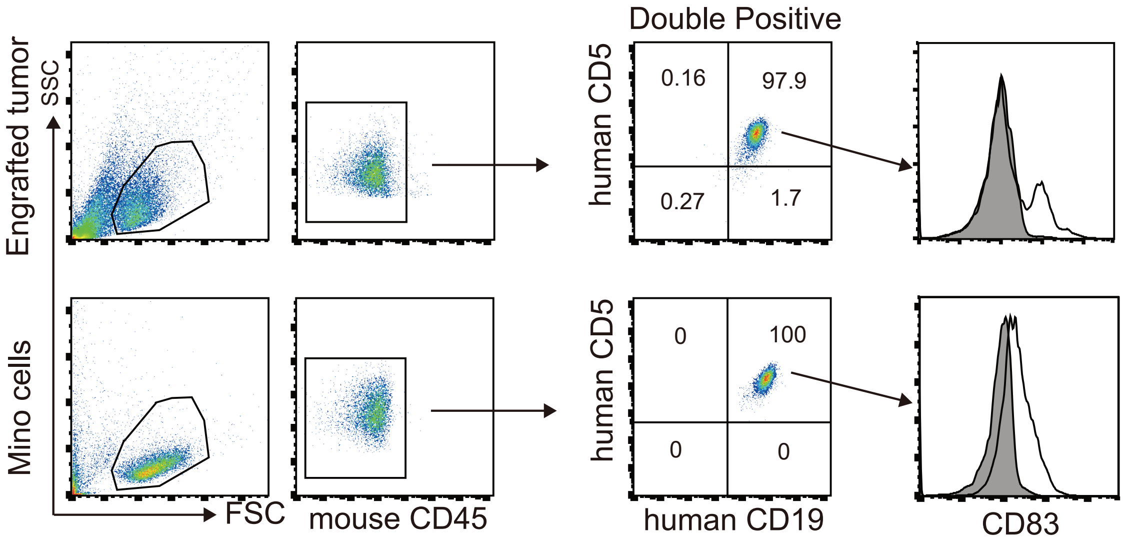

Supplementary figure 3

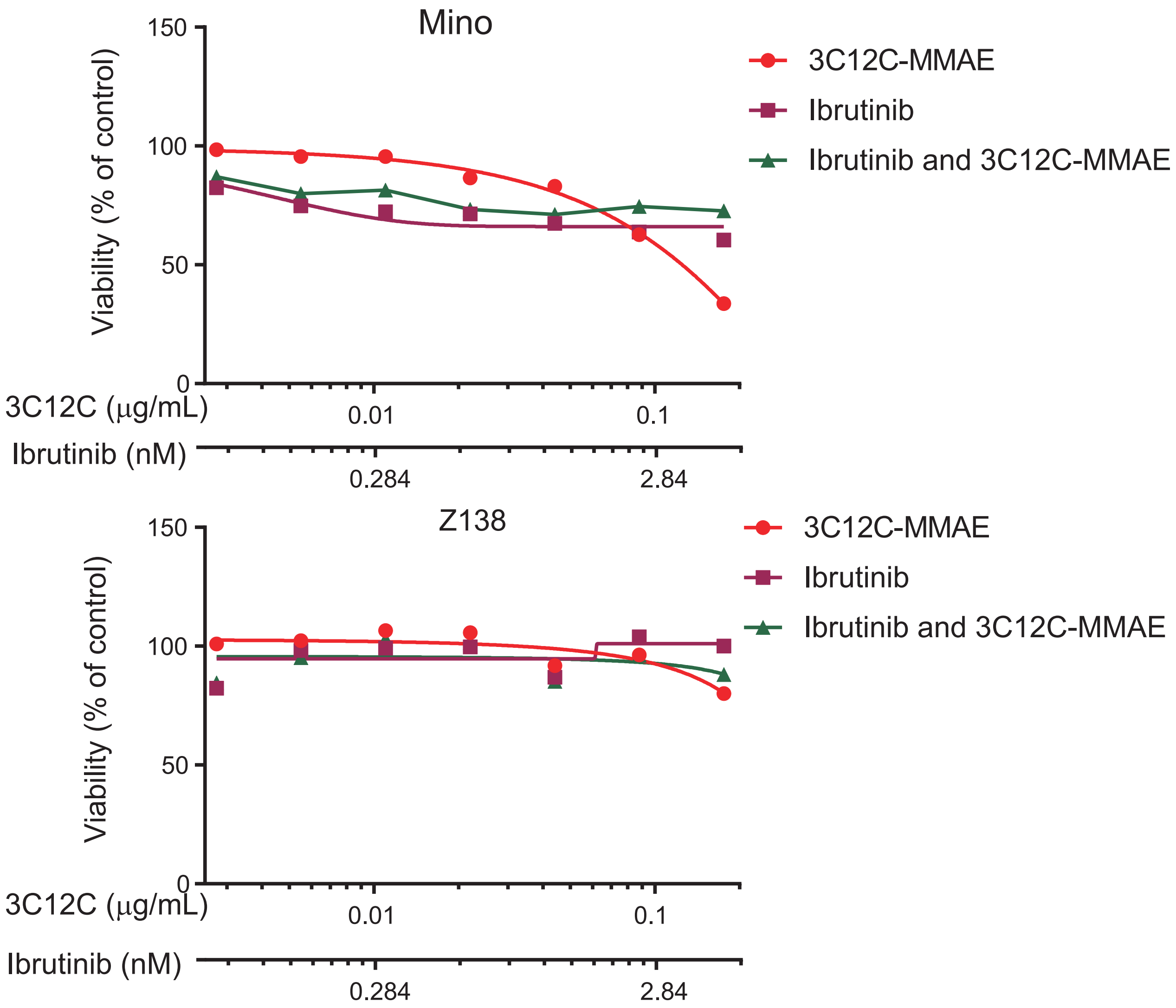

Supplementory figure 4

a

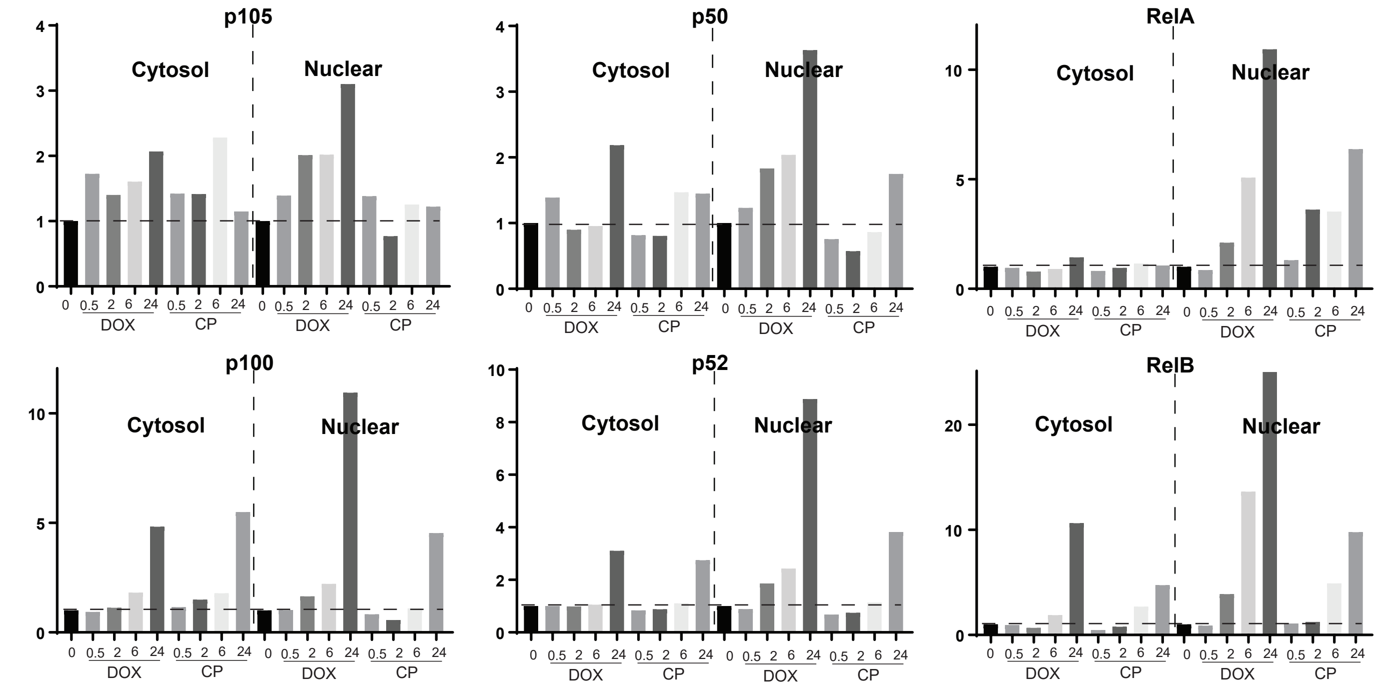

b

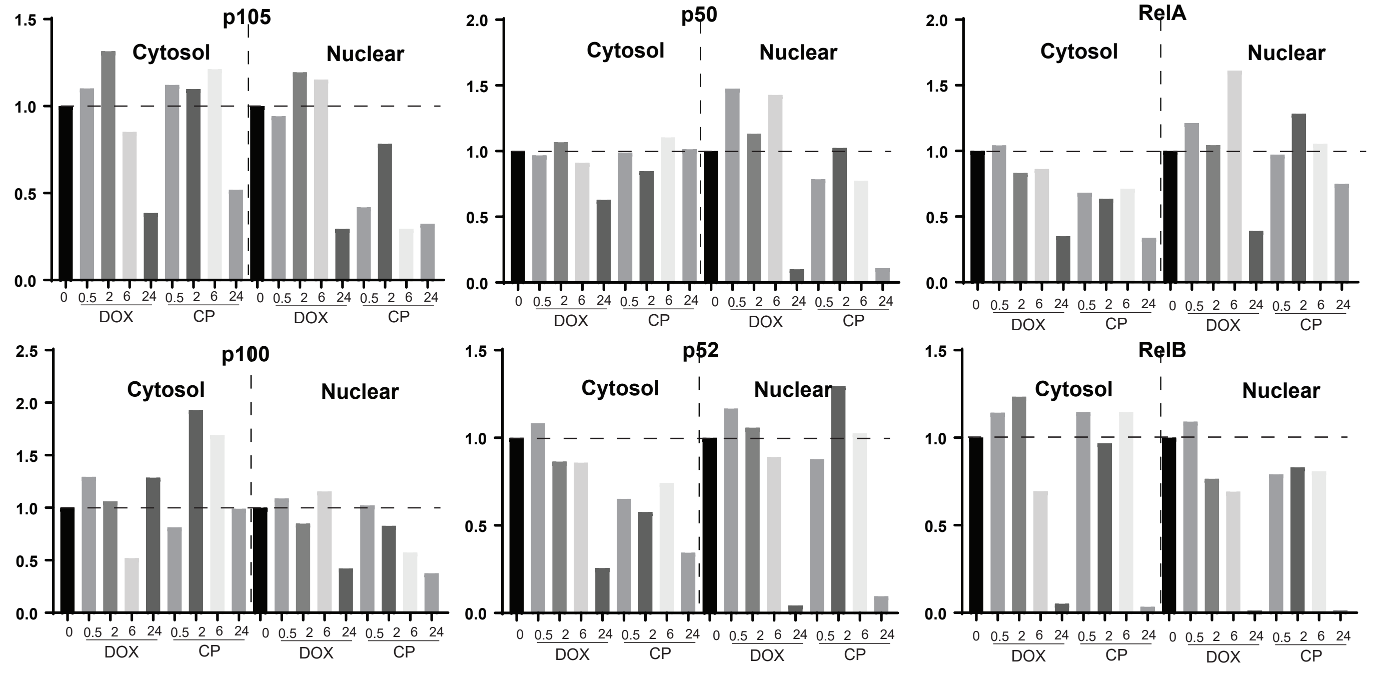

Supplementary figure 5

Supplementary figure legend

Supplementary figure 1: Human anti-human CD83 mAb 3C12C killed $CD83^+$MCL cell lines via ADCC.

Target cells MCL lines Mino, Rec-1 labelled with Calcein-AM were co-cultured with effector cells (human PBMC) at E:T ratio of 25:1 with increasing 3C12C concentration from 0 µg/mL to 5µg/ml at 37ºC for 3 hours. Supernatant was collected for fluorescence reading (excitation 485nm, emission 538nm) of released Calcein. ADCC activity was calculated (*n* = 3).

Supplementary figure 2: Sensitivity of MCL cell lines to the MMAE.

$CD83^+$ KM-H2, Mino, Rec-1 or $CD83^-$ Z138, Jvm2 cells were cultured with different concentrations of MMAE for 72 hours before determining viable cells by CellTiter-Glo Luminescent Cell Viability assay. Data from one representative experiment (*n* = 2) with half maximal inhibitory concentration (IC50) shown.

Supplementary figure 3: CD83 expression on the tumour cells from MCL xenograft mice.

Tumor was dissected from MCL engrafted mice at day 6 and tumor cells were isolated for analysing the CD83 expression. Representative flow cytometry plot showing gating strategy (upper) on mouse $CD45^-$ human $CD19^+$ human $CD5^+$ cells and CD83 staining (right) of the engrafted tumor cells. Mino cells in culture was used as control (bottom).

Supplementary figure 4: The combination effect of Ibrutinib and 3C12C-MMAE on the killing of Mino or Z138 cells.

Mino or Z138 cells were cultured with serially diluted 3C12C-MMAE (0.176, 0.088, 0.044, 0.022, 0.011, 0.0055, 0.00275 μg/mL), Ibrutinib (5, 2.5, 1.25, 0.625, 0.312, 0.156, 0.078 nM) or the combination of 3C12C-MMAE/Ibrutinib (0.176/5, 0.088/2.5, 0.044/1.25, 0.022/0.625, 0.011/0.312, 0.0055/0.0.156, 0.00275/0.078) for 72 hours. CellTiter-Glo Luminescent cell viability assay was used to determine the killing effect. Data were from one of three independent experiments.

Supplementary figure 5: Relative expression level of NF-κB signaling molecules in Mino and Z138 cells treated with Doxorubicin or Cyclophosphamide.

Mino or Z138 cells were cultured in the presence of Doxorubicin (DOX, 0.2ug/mL) or Cyclophosphamide (CP, 0.5mg/mL) for 30 minutes, 2 hours, 6 hours and 24 hours. Cytoplasmic and nuclear protein were isolated. Immunoblot analysis of cell lysate was performed with anti-NF-κB antibodies. The relative protein expression level was analysed with Image Lab 4.1 software (Bio-Rad).

Supplementary table: Chemotherapy reagents on CD83 expression of mantle cell lymphoma cell lines

**Chemotherapy reagents**	**Effect on CD83 expression**
Doxorubicin	Yes
Cyclophosphamide	Yes
Cytarabine	No
Vincristine	No
Temsirolimus	No
Lenalidomide	No
Bendamustine	No
